# Right Angle Percutaneous Endoscopic Gastrostomy Tube Extension to Prevent Tube-Induced Jejunoduodenal Intussusception

**DOI:** 10.14309/crj.0000000000000166

**Published:** 2019-08-15

**Authors:** Elliot Klein, Stephen Mularz, Seth Lapin

**Affiliations:** 1 Department of Gastroenterology, NYC Health + Hospitals, Coney Island Hospital, Brooklyn, NY

## CASE REPORT

**Figure 1.**
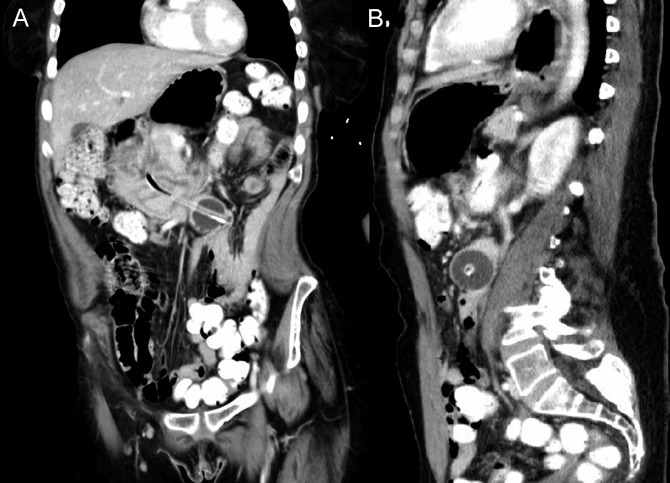
(A) Coronal and (B) sagittal abdominal computed tomography demonstrating retrograde jejunoduodenal intussusception with balloon migration into the second portion of the duodenum.

**Figure 2.**
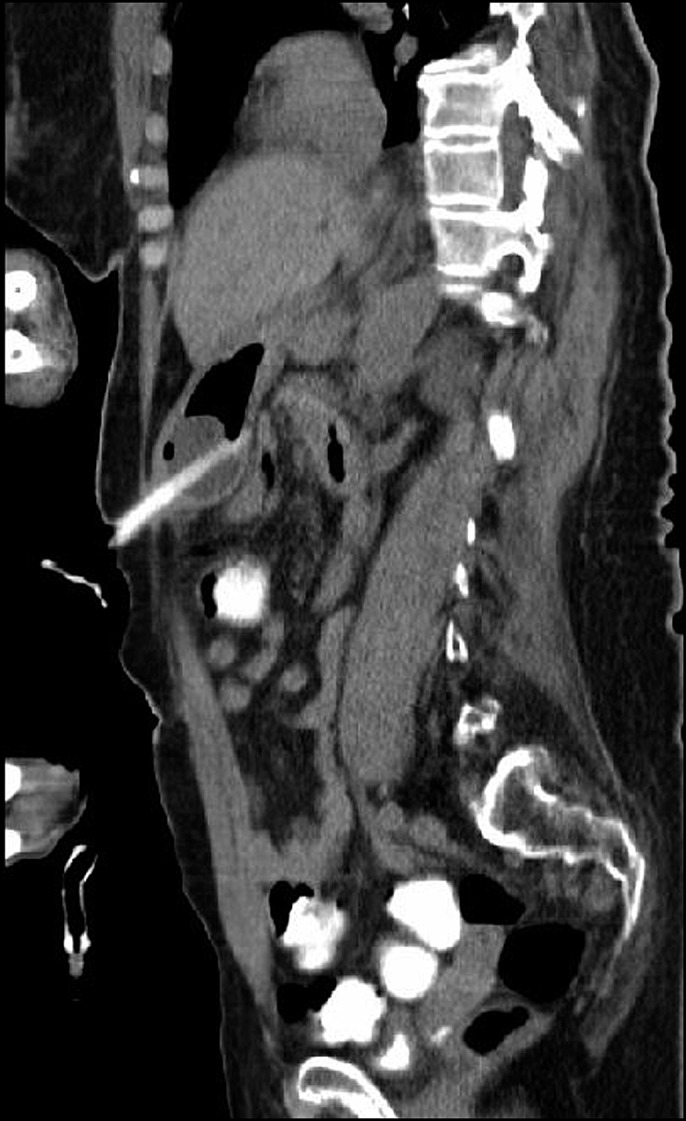
Resolution of intussusception with anchorage of balloon against anterior stomach wall.

A 62-year-old woman with a history of cerebral palsy, dementia, dysphagia, cerebral vascular accident, and 18 months of percutaneous endoscopic gastrostomy (PEG) tube placement presented from a skilled nursing facility with multiple episodes of nonbloody, bilious emesis, fever, and hypotension. Gastric contents were aspirated from the PEG tube and abdominal distention was noted. Hemogram demonstrated an elevated white blood cell count of 31.5 K/mm^3^ with a left-band shift and a lactic acid level of 4.9 mmol/L. Abdominal computed tomography (CT) with contrast administered via PEG displayed the first and second portions of the duodenum to be thickened with evidence of retrograde jejunoduodenal intussusception due to tube migration into the left upper abdominal quadrant (Figure 1). The PEG was initially evaluated and repositioned at bedside with deflation of the bumper and gentle retraction of the tube back toward the stomach. Repeat migration was of concern as the tube altered position over the following days. Later, the tube was replaced with a 20 French right-angle PEG with position confirmed via plain film. Repeat CT demonstrated resolution of intussusception with maintained thickening of the proximal duodenal wall (Figure 2). Another CT scan performed 9 months later indicated continued proximal duodenal wall thickening likely due to scarring from the intussusception inflammatory process. Endoscopic evaluation was not performed to evaluate the duodenal wall thickening as multiple comorbidities, including severe dementia and mental retardation, precluded the patient to a change in management.

Retrograde jejunoduodenal intussusception is a very rare complication of gastrostomy tube use. Bowel ischemia is of grave concern requiring a high level of suspicion in patients presenting with nausea and vomiting and a history of PEG tube. The mechanism of intussusception is not well understood, but several hypotheses have been previously discussed.^1^ Fundamentally, migration of the tube due to peristalsis, with subsequent fixation to the bowel wall, allows for retrograde jejunal invagination over the balloon.^2,3^ This may be further exacerbated by attempts to reposition the tube without balloon deflation causing retraction toward the pylorus.^4^ The use of external fixation devices seek to immobilize the balloon but has proven to be inadequate in some patients. Here, we recommend the use of a right-angle tube to further restrict movement. The right-angle tube was employed because the tube structure allows it to be less obtrusive, while the angulation anchors the tube against the external abdomen, thereby reducing inward migration. Low-profile tubes were not available in our facility as the demographic primarily attended to by our institution is nonpediatric and unconcerned with aesthetics. Additionally, carrying a full array of low-profile tubes is often costly for smaller facilities. Although rare, prevention of migration with resulting intussusception should be considered in patients with a history of recurrent malposition and may be reinforced by the advantages of utilizing a right-angle PEG.

## DISCLOSURES

Author contributions: E. Klein and S. Mularz reviewed the literature and wrote the manuscript. S. Lapin edited the manuscript and is the article guarantor.

Financial disclosure: None to report.

Informed consent was obtained from the patient's proxy for this case report.
